# Impact of larval and cocoon burial depth on emergence of adult soybean gall midge (Diptera: Cecidomyiidae)

**DOI:** 10.1093/jisesa/ieag024

**Published:** 2026-03-11

**Authors:** Isak J Stillwell Jardine, James P Menger, Arthur V Ribeiro, Robert L Koch

**Affiliations:** Department of Entomology, University of Minnesota, Saint Paul, MN, USA; Department of Entomology, University of Minnesota, Saint Paul, MN, USA; Department of Entomology, University of Minnesota, Saint Paul, MN, USA; Department of Entomology, University of Minnesota, Saint Paul, MN, USA

**Keywords:** cultural control, integrated pest management, larval dispersal, soil ecology

## Abstract

Soybean gall midge, *Resseliella maxima* Gagné, is a pest of soybean that causes severe yield loss, with no specific management tactics currently being widely implemented. Due to *R. maxima* forming cocoons and pupating in soil, characterizing its cocooning behavior and studying the effects of artificial burial on adult emergence may reveal possible cultural control tactics like tillage. *R. maxima* larvae were released and allowed to pupate in vials filled with sand, which were then dissected into cross-sections to identify the depth at which cocoons were formed. *R. maxima* tended to form cocoons within the first 1 cm of sand when 10 larvae were released, and within the first 1.5 cm of sand when 50 larvae were released. In a second experiment, cocoons were buried at depths up to 3 cm in 0.5 cm increments. When cocoons were buried, adult emergence decreased and was delayed as depth increased, with no adult emergence when cocoons were buried deeper than 1.5 cm. In a third experiment, larvae were buried at depths up to 12 cm in 1 cm increments. When larvae were buried, adult emergence decreased and was delayed as depth increased; however, there was emergence from the deepest tested depth. Upward movement of larvae plateaued and decreased as burial depth increased, with greater burial depths also associated with lower cocooning rates. These findings suggest that burial of cocooned *R. maxima* can effectively reduce adult emergence, and that tillage should be explored as a potential management tactic to control this pest.

## Introduction

Soybean gall midge, *Resseliella maxima* Gagné (Diptera: Cecidomyiidae), emerged as a pest of soybean in 2018 in the midwestern United States (Gagné et al. 2019). However, it remains unknown if *R. maxima* is a native species that underwent a host range expansion to now include soybean, or an invasive species that has remained undocumented elsewhere in the world (Dean and Hodgson 2022). As of 2025, *R. maxima* has been found in 185 counties across 7 midwestern states (Iowa, Kansas, Minnesota, Missouri, North Dakota, Nebraska, and South Dakota) (Soybean Gall Midge Alert Network 2025). Adults of *R. maxima* oviposit in naturally occurring fissures or cracks in soybean stems, and larvae feed on tissue within the stem (McMechan et al. 2021). Larval feeding damages vascular tissue, disrupting the flow of water and nutrients in the stem, leading to girdling and death of plants (McMechan et al. 2021). Soybean fields infested with *R. maxima* may experience yield losses throughout the field, but losses are greatest on field edges (McMechan et al. 2021). *R. maxima* pupates in cocoons in the soil and undergoes 2 or more generations per year, with later-season larvae overwintering in the soil as larvae in cocoons and emerging the following spring (Dean and Hodgson 2022).

Soybean, *Glycine max* (L.) Merrill (Fabales: Fabaceae) is a source of protein and seed oil and is the second most important crop in the United States, with over 35 million hectares planted nationwide generating $44 billion in 2024 (National Agricultural Statistics Service 2025). Minnesota is the fourth largest producer of soybeans in the country, with 2.9 million hectares planted in 2024 (National Agricultural Statistics Service 2025). The 6 other states where *R. maxima* has been found all rank among the top 11 soybean producing states in the United States (National Agricultural Statistics Service 2025). The economic importance of soybeans in the Midwest coupled with the severe adverse effects of *R. maxima* infestation makes finding effective management strategies important.

Few effective management tactics for *R. maxima* have been identified thus far. Foliar- and seed-applied formulations of insecticides have shown some efficacy at protecting soybean from *R. maxima* but are likely not sufficient for standalone control of this pest (Hodgson and Helton 2021, Montenegro et al. 2022, Cooper and McMechan 2024, Umezu and McMechan 2025). In contrast, a soil application of the insecticide phorate at planting was found to reduce the abundance of *R. maxima* larvae and plant injury as well as improve yield; however, this tactic requires specialized equipment for application which limits its implementation (McMechan 2021).

Nonchemical tactics like biological and cultural control of *R. maxima* are under evaluation. Two species of *Synopeas* (Hymenoptera: Platygastridae) have been identified as parasitoids of *R. maxima*, but further work is needed to characterize the potential for these parasitoids to provide pest suppression across the region (Melotto et al. 2023a, 2023b, von Gries et al. 2025a). *Pterostichus melanarius* (Illiger) (Coleoptera: Carabidae) has been examined as an abundant and potentially voracious predator of *R. maxima*, but its predation rate on this pest in the field is unknown (Melotto et al. 2023c, von Gries et al. 2025b). Several other generalist predators are in the early stages of being explored as possible biological control agents of *R. maxima* as well (Melotto et al. 2023c). Hilling, a form of cultural control in which soil is piled around the base of the plant stems to prevent oviposition, has been shown to be an effective cultural control tactic for reducing *R. maxima* infestation and plant injury which corresponded to increased yield; however, hilling requires specialized equipment and is not commonly used as a cultural control tactic in soybean (McMechan et al. 2023, Gupta and McMechan 2025). These tactics show promise, but there is need for further investigation of alternative management strategies for *R. maxima*.

Some ground-dwelling insect pests, especially those that overwinter in the soil, can be managed with conventional tillage methods through both mechanical damage to the insect as well as burial of the insect at deeper depths, which prevents their emergence (Matlock et al. 2017, Rowen et al. 2020). Pupation in the soil is relatively common across Cecidomyiidae (Gagné 2024), and a lower adult emergence with increasing burial depth of larvae has been documented for some species within this family (Chapin et al. 1992, Franzmann et al. 2006, Chen and Shelton 2007). For example, some forms of tillage are effective at reducing populations of the Hessian fly, *Mayetiola destructor* Say (Diptera: Cecidomyiidae) (Chapin et al. 1992). Similar responses to tillage have also been observed for other pests that pupate in the ground (Wichura et al. 2022). However, aspects of the biology of *R. maxima* related to burial of immatures and subsequent emergence of adults are still unknown. We hypothesize that adult emergence will decrease with increasing burial depth of *R. maxima* larvae and cocoons. This study was conducted with the objectives of (i) characterizing the burial behavior of *R. maxima* and (ii) characterizing the effect of artificial burial of *R. maxima* larvae and cocoons on adult emergence.

## Materials and Methods

### Insect Colony

*R. maxima* used in the experiments described below were obtained from a laboratory colony maintained at the University of Minnesota, Saint Paul, Minnesota, United States. The *R. maxima* originated from Minnesota and had been maintained under laboratory conditions (25 °C, 16:8 light:dark cycle, and 70% relative humidity). In the colony, larvae were reared on soybean variety “Sheyenne” grown in small pots (8.9 × 8.9 × 8.9 cm) containing germinating potting mix (sphagnum peat, coarse perlite, and vermiculite) (PRO-LINE C/GP Germinating Mix, Jolly Gardener, Poland, Maine, United States). Mature larvae were extracted from soybean stems and transferred to polystyrene vials (25 mm outer diameter, 95 mm height; Flystuff Drosophila Vials, Genesee Scientific, San Diego, California, United States) containing moistened sand for pupation. This type of vial was used for all experiments described below. Emerged adults were released into cages containing potted soybean for mating and oviposition.

For the experiments described below, presumed third instar *R. maxima* were distinguished by their relatively large size and bright orange coloration (Gagné et al. 2019). Cocoons used in the experiments described below were produced by placing mature larvae into vials with sand as described above and were extracted from the sand after 8 d by carefully rinsing the sand away with a stream of water from a squirt bottle. Preliminary experiments showed that 8 d was adequate time for most *R. maxima* larvae to form cocoons and pupate.

### Cocooning Depth

Vials were filled with 21 g of dry sand, which totaled a height of about 3 cm of sand in the vials. Sets of 10 or 50 larvae were placed on the surface of the sand in the vials. The sand was moistened by adding 3.4 ml of water to each vial. Each larval density (10 or 50 larvae) was replicated in 8 vials. Vials were capped with foam vial plugs (Droso-Plugs Drosophila Closures, Genesee Scientific, San Diego, California, United States), leaving 4 cm of headspace between the surface of the sand and the bottom of the plug, and placed in growth chambers (25 °C, 16:8 light:dark cycle, and 70% relative humidity). After 8 d, each vial was carefully inverted over a plastic dish to remove the contents of the vial as one unbroken column of sand. Each sand column lying on its side was dissected with a razor blade into 0.5-cm cross-sections, descending sequentially from what was the upper surface of the sand in the vial. Each cross-section was then individually rinsed with a slow stream of water from a squirt bottle to separate *R. maxima* from the sand. *R. maxima* cocoons, as well as any larvae or pupae outside of cocoons, were counted and recorded for each cross-section. Cocoons and pupae outside of cocoons were grouped together as successfully cocooned, as formation of a cocoon occurs before pupation.

### Emergence of Adults after Burial of Cocoons

Vials as described above were set up with depth treatments of 0.5-cm increments of sand ranging from 0 to 3 cm (7 depth treatments total, with 1 treatment per vial). For the creation of the depth treatments, sand depth was measured by weight with 0.5 cm of dry sand weighing an average of 2.97 g. Treatments differed in the height of the base layer of sand added to the vials prior to addition of the *R. maxima* cocoons. The base layer of sand ranged from 2 cm of sand for the 3-cm depth treatment to 5 cm for the 0-cm treatment. After the appropriate base layer of sand was added to a vial, 10 cocoons were placed on the surface of the base layer of sand and then the corresponding top layer of sand was added on top of the cocoons to bring the total column of sand in the vial to 5 cm. The sand was moistened by adding 4.5 ml of water to each vial. Each depth treatment was replicated 12 times. Vials were capped with foam vial plugs, leaving 2 cm of headspace between the surface of the sand and the bottom of the plug, and placed in growth chambers (25 °C, 16:8 light:dark cycle, and 70% relative humidity). Vials were checked daily and any emerged adults were collected, counted and sexed. Nine d after vials were set up (ie after adult emergence ceased), the sand columns from vials were dissected as described above to assess the location and life stage of remaining unemerged *R. maxima*.

### Emergence of Adults after Burial of Larvae

Preliminary experiments indicated that this assessment would require maximum sand depths deeper than what a standard vial could hold, so deeper vials (double-height vials) were created. To do so, the base of a vial was sanded down with 100-grit sandpaper until the base could be easily removed. The top of an intact vial was attached to the top of a sanded vial using clear packing tape, ensuring that no gaps were present so that vials would not leak, lose humidity or allow larvae to escape throughout the course of the experiment.

The resulting double-height vials were set up with depth treatments of 2-cm increments ranging from 0 to 12 cm (7 depth treatments total) of sand. Sand was measured by weight as described above. The base layer of sand ranged from 2 cm of sand for the 12-cm depth treatment to 14 cm for the 0-cm treatment. After the appropriate base layer of sand was added to a vial, 10 larvae were placed on the surface of the base layer of sand and then a corresponding top layer of sand was added on top of the larvae to bring the total column of sand in the vial to 14 cm. Vials were capped with foam vial plugs, leaving 3 cm of headspace between the surface of the sand and the bottom of the plug, and placed in growth chambers (25 °C, 16:8 light:dark cycle, and 70% relative humidity). The vials were randomly allocated to 2 separate subsets with 12 replications of each depth treatment in each subset. One subset was maintained for adult emergence, and the other subset was dissected before adult emergence.

The subset of vials designated to be dissected before adult emergence were dissected 8 d after introduction of larvae. The double-height vials were disassembled before dissection by cutting the sand column at the joining point between the vials. The sand columns from the detached half double-height vials were dissected as described above into 1-cm cross-sections. *R. maxima* larvae, cocoons, and pupae outside of cocoons were counted and recorded for each cross-section.

The subset of vials designated to be left for adult emergence were checked at 24-h intervals and any emerged adults were collected, counted and sexed. The sand columns from vials were dissected as described above 29 d after being set up to assess the location and life stage of remaining unemerged *R. maxima*.

### Statistical Analysis

All analyses were performed and figures prepared using R Core Team (2025) and Posit Team (2025). For the cocooning depth experiment, the proportion of *R. maxima* cocoons and pupae outside of cocoons successfully retrieved was analyzed using a mixed-effects binomial generalized linear model (Package, code: lme4, glmer; Bates et al. 2015) with depth of retrieval and the initial number of larvae as fixed factors. Because larvae released in each vial could potentially be retrieved at any depth in the sand column, vial was also included as a random factor to account for the lack of independence among depths within the same vial. Depths with no emergence were removed from the analysis. Model fit was assessed using diagnostic plots (quantile-quantile and predicted against scaled residuals), as well as goodness-of-fit tests on the scaled residuals for uniformity, presence of outliers and over/underdispersion (DHARMa, simulateResiduals; Hartig et al. 2024). Significance of fixed effects were estimated using type II Wald *χ*^2^ tests (car, Anova; Fox and Weisberg 2019) followed by multiple pairwise comparisons of estimated marginal means (emmeans, emmeans; Lenth et al. 2025). The proportion of cocoons and pupae outside of cocoons retrieved at depths included in the model were also formally compared to depths with no emergence by comparing their estimated marginal means to zero using *t*-tests (emmeans, summary; Lenth et al. 2025).

The proportion emergence of adults from the assays testing burial of cocoons or larvae were analyzed separately using binomial generalized linear models (stats, glm; R Core Team 2025) with a mean bias-reduction adjustment (brglm2, brglm_fit in the “methods” argument of the glm function; Kosmidis et al. 2020) to account for complete separation of the data. Burial depth, sex of emerged adults, and their interaction were included in each model as fixed factors. Model fit was assessed using diagnostic plots and goodness-of-fit tests on the scaled residuals, significance of fixed effects was estimated, and multiple pairwise comparisons were obtained using the methods described above.

The cumulative proportion of emerged adults over time from the assays testing burial of cocoons or larvae were analyzed separately using truncated (ie lower limit fixed at 0 and upper limit fixed at 1) time-to-event models (drcte, drmte; Onofri et al. 2022). Combinations of treatment (ie burial depth) and sex were initially included in each model, but male and female data were pooled in the final models because of the overall lack of differences between sexes in the cumulative proportion of emerged adults at each depth. Models were fit with the log-logistic, log-normal, Weibull type 1, or Weibull type 2 cumulative distribution functions (CDFs), and the model with the lowest Akaike information criteria value was selected. In the final models (ie pooled males and females), the log-logistic CDF (Equation [1]) and Weibull type 1 CDF (Equation [2]) were selected for the burial of cocoons assay and burial of larvae assay, respectively:


(1) P(t)=1/(1+exp(−b×(log(t)−log(e))))

(2) P(t)=exp(−exp(b×(log(t)−log(e))))

where *t* is the time, *P*(*t*) is the proportion of emerged adults at time *t* in days, *b* is the slope at the inflection point *e* (ie at the time of 50% emergence). Significance of treatments (ie burial depth, or the combination of burial depth and sex for the initial models) was estimated for each model with a likelihood-ratio *χ*^2^ test (drcte, compCDF; Onofri et al. 2022), and time to 50% emergence were compared among treatments using 1-sample *t*-tests for the difference between each pairwise comparison (drcte, compParmte; Onofri et al. 2022). Confidence intervals of estimates of slopes and SEs of estimates of time to 50% emergence were obtained using cluster-robust estimations using sandwich estimation quantile (drcte, quantile; Onofri et al. 2022). Due to low or no emergence, treatments (ie burial depth) greater than 1 cm for the burial of cocoons assay and greater than 6 cm for the burial of larvae assay were not included in the analyses.

The mean larval movement within the sand column from the burial of larvae assay was analyzed using a mixed-effects linear model (nlme, lme; Pinheiro and Bates 2000) with burial depth as a fixed factor and vial as a random factor to account for the lack of independence among depths. Additionally, the variance structure of the model was allowed to vary among treatments (ie burial depth) to account for heteroscedasticity of residuals (nlme, varIdent (form = ∼1|Treatment) in the “weights” argument of the lme function; Pinheiro and Bates 2000). Model fit was assessed using diagnostic plots of linearity and normality of residuals (performance, check_model; Lüdecke et al. 2021). Significance of fixed effect and multiple pairwise comparisons were obtained using the methods described above for the cocooning depth experiment.

The proportion of larvae cocooned from the burial of larvae assay was analyzed using a mixed-effects binomial generalized linear model (lme4, glmer; Bates et al. 2015) with depth of retrieval as a fixed factor. Because larvae released in each vial could potentially be retrieved at any depth in the sand column, vial was also included in the model as a random factor to account for the lack of independence among depths within the same vial. Model fit was assessed using diagnostic plots and goodness-of-fit tests on the scaled residuals, significance of fixed effect was estimated, and multiple pairwise comparisons were obtained using the methods described above for the cocooning depth experiment.

## Results

### Cocooning Depth

**Fig. 1.**
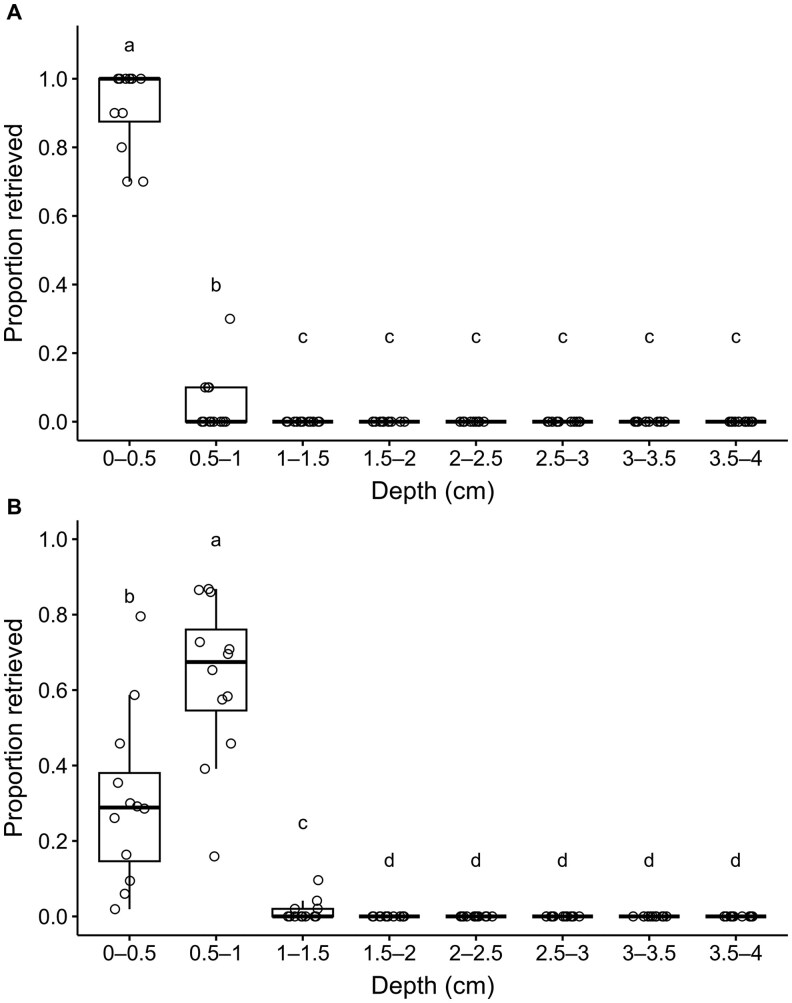
Proportion of *Resseliella maxima* cocoons and pupae outside of cocoons retrieved by depth after 10 larvae (A) or 50 larvae (B) were released onto the surface of sand in vials and allowed to develop for 8 d.

All individuals (100%) were recovered when 10 larvae were released per vial. On average, 96.5% of individuals were recovered when 50 larvae were released per vial. The proportion of retrieved cocoons and pupae outside of cocoons differed significantly among depths (*χ*^2^ = 218.07, *df*  =  2, *P* <0.001) and was significantly affected by the initial number of larvae (*χ*^2^ = 5.31, *df*  =  1, *P =* 0.02) and the interaction between depth and the initial number of larvae (*χ*^2^ = 153.21, *df*  =  1, *P* <0.001) (Fig. 1). When 10 larvae were released per vial, the proportion of retrieved cocoons and pupae outside of cocoons was highest at 0 to 0.5 cm, followed by 0.5 to 1 cm, but no cocoons were present from 1 to 4 cm (Fig. 1A). When 50 larvae were released per vial, the proportion of retrieved cocoons and pupae outside of cocoons was highest at 0.5 to 1 cm, intermediate at 0 to 0.5 cm, and lower at 1 to 1.5 cm, but no cocoons were present from 1.5 to 4 cm (Fig. 1B).

### Emergence of Adults after Burial of Cocoons

**Fig. 2.**
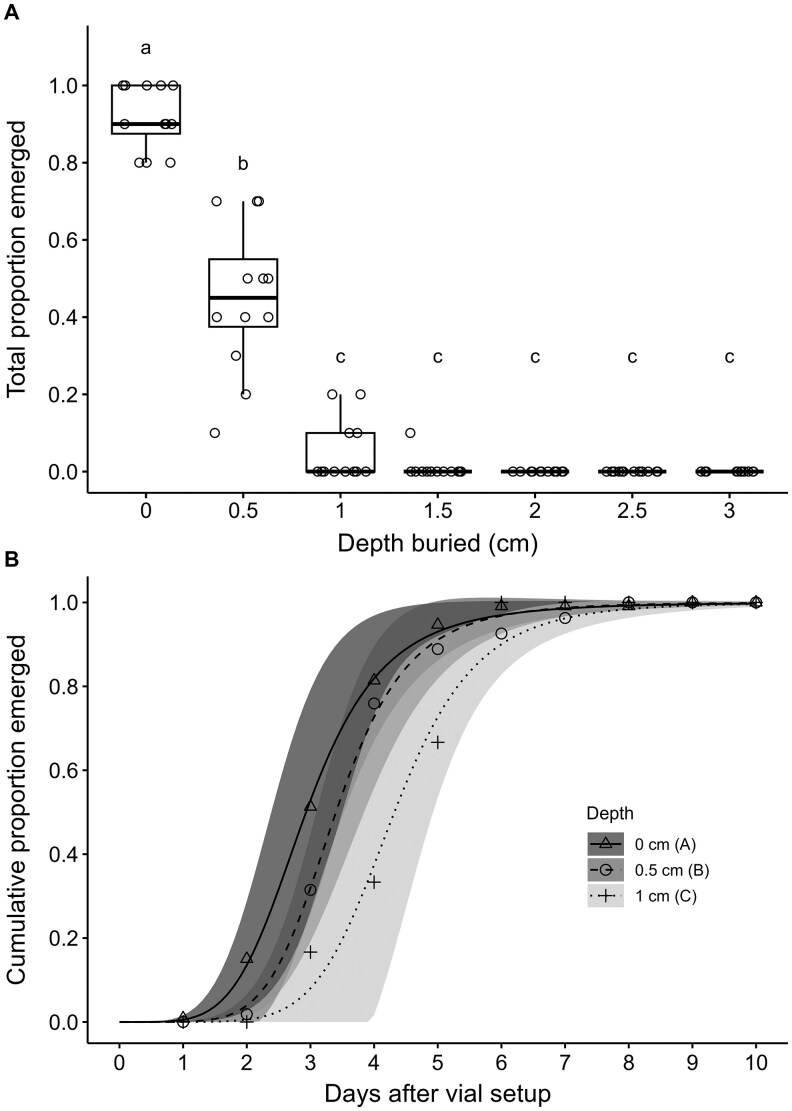
Total proportion of emerged adults (A) and cumulative proportion of emerged adults over time (B) after burial of *Resseliella maxima* cocoons at different depths in vials of sand. Cumulative emergence over time from depths greater than 1 cm were not included in analysis, due to very low or no emergence.

The overall mean proportion emergence of adults was significantly affected by burial depth of cocoons (*χ*^2^ = 453.64, *df*  =  6, *P* <0.001), but not by sex of emerged adults (*χ*^2^ = 0.73, *df*  =  1, *P =* 0.39), nor by the interaction of sex and burial depth (*χ*^2^ = −5.90, *df*  =  6, *P* = 1.00) (Fig. 2A). Adult emergence decreased as burial depth increased, with no emergence observed for burial depths greater than 1.5 cm (Fig. 2A). The mean proportion emergence was highest for cocoons at 0 cm, intermediate for those buried at 0.5 cm, and lowest for those buried from 1 to 3 cm, with only burial depths of 1 and 1.5 cm showing emergence in this range (Fig. 2A).

The time to 50% emergence of adults did not significantly differ between sexes when cocoons were buried at 0 cm (*t*  =  1.59, *P =* 0.11) or 0.5 cm (*t*  =  −0.63, *P =* 0.53), with burial depths at or greater than 1 cm not included in this analysis due to very low emergence of adults. When sexes were pooled, cumulative emergence of adults over time differed significantly among burial depths (*χ*^2^ = 11.97, *df*  =  2, *P =* 0.02). The time to 50% emergence of pooled adults was shortest for cocoons at 0 cm (2.9 ± 0.3 d), intermediate for those buried at 0.5 cm (3.4 ± 0.2 d), and longest for those buried at 1 cm (4.0 ± 0.3 d) (Fig. 2B).

### Emergence of Adults after Burial of Larvae

**Fig. 3.**
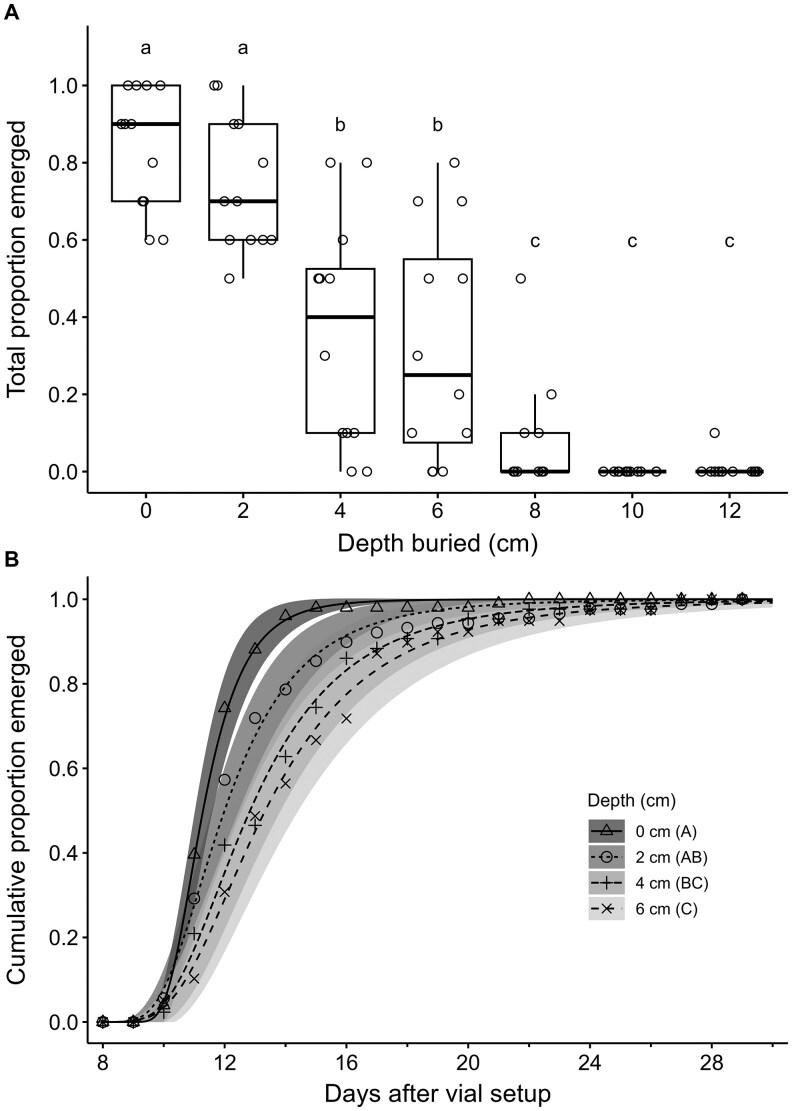
Total proportion of emerged adults (A) and cumulative proportion of emerged adults over time (B) after burial of *Resseliella maxima* larvae at different depths in vials of sand. Cumulative emergence over time from depths greater than 6 cm was not included due to low or no emergence.

The overall mean proportion emergence of adults was significantly affected by burial depth of larvae (*χ*^2^ = 350.74, *df*  =  6, *P* <0.001) and sex of emerged adults (*χ*^2^ = 11.06, *df*  =  1, *P* <0.001), but not by the interaction of sex and burial depth (*χ*^2^ = 1.12, *df*  =  6, *P =* 0.98) (Fig. 3A). The mean proportion emergence across burial depths was highest for females (0.19 ± 0.03) compared to that of males (0.14 ± 0.02). Across sexes, adult emergence decreased as burial depth increased, with mean proportion emergence highest for larvae at 0 and 2 cm, intermediate for those buried at 4 and 6 cm, and lowest for those buried from 8 to 12 cm (Fig. 3A). No emergence was observed for those buried at 10 cm, but 1 female emerged from those buried at 12 cm (Fig. 3A).

The time to 50% emergence of adults significantly differed between sexes for larvae at 0 cm (*t*  = 1.97, *P =* 0.049) and 2 cm (*t*  =  2.23, *P =* 0.03), but not for those buried at 4 cm (*t*  =  0.78, *P =* 0.44) or 6 cm (*t*  =  1.22, *P =* 0.22). Burial depths greater than 6 cm were not included in this analysis due to very low emergence. When sexes were pooled, cumulative emergence of adults over time differed significantly among burial depths (*χ*^2^ = 54.22, *df*  =  6, *P* <0.001) (Fig. 3B). Time to 50% emergence of pooled adults increased with burial depth, being significantly shorter at 0 cm (11.3 ± 0.1 d) than at 4 cm (12.8 ± 0.5 d) or 6 cm (13.3 ± 0.7 d) (Fig. 3B). In addition, time to 50% emergence of pooled adults at 2 cm (12.0 ± 0.4 d) was shorter than that of 6 cm (Fig. 3B).

**Fig. 4.**
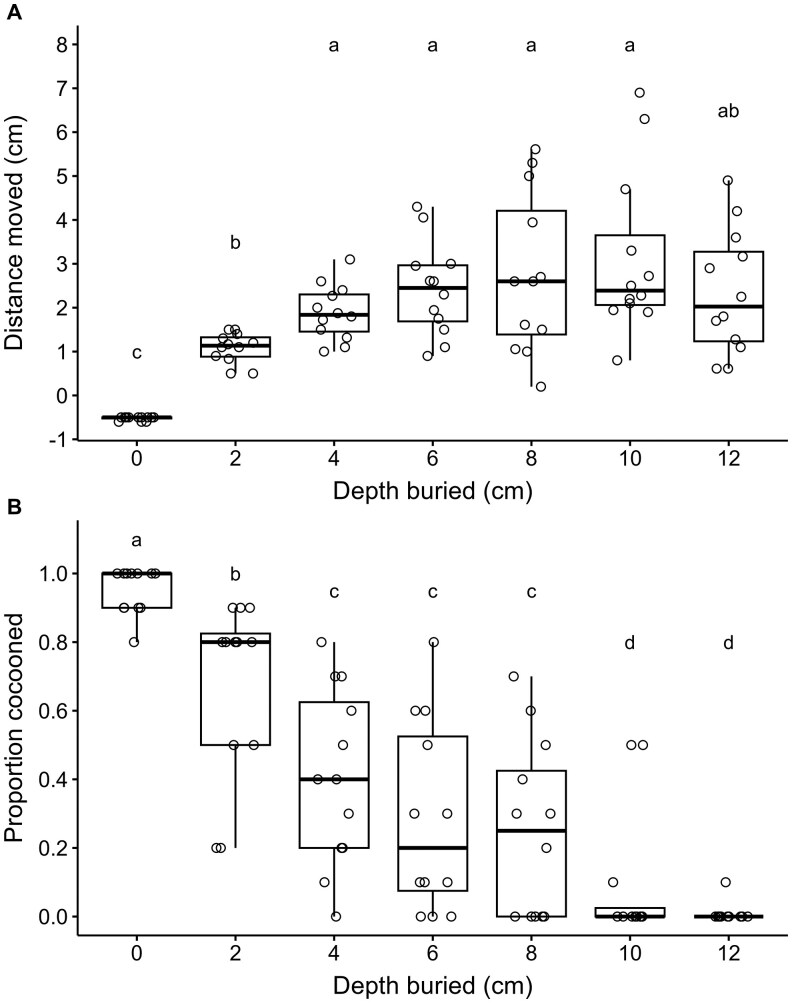
Mean vertical distance moved (i.e., distance between burial depth and retrieval depth) within the sand column (A) and proportion of individuals that were cocooned at time of retrieval (B) after burial of *Resseliella maxima* larvae at different depths in vials with sand.

The mean vertical larval movement within the sand column over 29 d was significantly affected by burial depth (*χ*^2^ = 659.94, *df*  =  6, *P* <0.001) (Fig. 4A). Mean larval movement was lowest for those buried at 0 cm, intermediate for those buried at 2 cm, and highest for those buried from 4 to 10 cm; however, larval movement for those buried at 12 cm did not significantly differ from any burial depth except 0 cm (Fig. 4A).

The mean proportion of larvae cocooned over 29 d was significantly affected by burial depth (*χ*^2^ = 171.68, *df*  =  6, *P* <0.001) (Fig. 4B). The mean proportion of larvae cocooned was highest for larvae buried at 0 cm (Fig. 4B). Among the other burial depths, the mean proportion of larvae cocooned was highest for larvae buried at 2 cm, intermediate for those buried from 4 to 8 cm, and lowest for those buried at 10 and 12 cm (Fig. 4B).

## Discussion

This study represents the first examination of the effects of artificial burial of *R. maxima* cocoons and larvae on the emergence of adults. *R. maxima* is a novel and destructive pest of soybean that causes high yield loss in cases of severe infestation. Relatively little is known about its biology and life history, which hinders the implementation of management tactics to control *R. maxima* infestations.

After release of larvae on the surface of the sand, the depth at which cocoons formed was density dependent. Releasing 10 *R. maxima* larvae on the surface of the sand resulted in all larvae forming cocoons in the first 1 cm of sand, with a majority of cocoons being formed between 0 and 0.5 cm. However, releasing 50 *R. maxima* larvae on the surface of the sand resulted in a greater proportion of larvae forming cocoons between 0.5 and 1 cm with additional cocoons being formed down to 1.5 cm. Higher initial numbers of larvae may have resulted in cocoons being formed slightly deeper in the soil due to volume constraints within the narrow vial. Regardless of the number of larvae initially released, all cocoons were formed within the range of depths from which adults were able to emerge when cocoons were buried in the following experiment. Similarly, in laboratory assays, 92% of cocoons of *Resseliella* (*Thomasiniana*) *theobaldi* (Barnes) (Diptera: Cecidomyiidae) occurred within the top 1 cm of soil (Pitcher 1952). In addition, field sampling for *R. theobaldi* recovered 83%, 14%, and 3% of the pupae in the first, second, and third centimeters from the soil surface, respectively (Pitcher 1952). Under field conditions, soil cores collected from fields infested with *R. maxima* showed that 75.5% of cocoons were found within 2 cm of the surface, and 96.5% of cocoons were formed within 6 cm of the surface (da Silva Lima 2023). Deeper cocooning depths observed in the field could be due to differences between properties of soil in the field and the sifted sand used in this study, which could affect the digging behavior of *R. maxima* larvae. The pupation depths of both *Euxesta eluta* Loew and *Chaetopsis massyla* (Walker) (Diptera: Ulidiidae), were significantly affected by soil type and moisture levels (Allan 2023). However, the pupation depth of Swede midge, *Contarinia nasturtii* (Kieffer) (Diptera: Cecidomyiidae), was not significantly affected by soil type (Chen and Shelton 2007).

Burying cocoons of *R. maxima* deeper than 1.5 cm beneath the surface of the sand prevented emergence of *R. maxima* adults, with the 1.5 cm depth treatment having only 1 emerging adult across all replications. This pattern is similar to that observed in a study burying *C. nasturtii* cocoons, where no adults were able to emerge from cocoons buried deeper than 1 cm (Readshaw 1968). Observations of empty pupal exuviae on the surface of the sand in vials where cocoons had been buried suggest that *R. maxima* pupae may exit buried cocoons and move to the surface for adult emergence (Stillwell Jardine, personal observations). Such movement of pupae in the soil has also been observed for *R. theobaldi* (Pitcher 1952).

While burial of *R. maxima* larvae generally resulted in less emergence with increasing depths as well, the emergence of 1 adult at 12 cm, the deepest depth treatment in our study, shows that burial of larvae is less effective than burial of cocoons in preventing adult emergence. Larval movement of unemerged *R. maxima* showed that while larvae were able to move an average of about 3 cm upward, several were able to move much more. In contrast, negative larval movement in the 0 cm treatment indicated downward movement of the larvae, which was due to the larvae being released on the surface of the sand and digging down to pupate. As burial depth increased, *R. maxima* were less likely to have formed cocoons when they were retrieved upon dissection of sand vials, suggesting that larvae do not form cocoons until they are near the surface. Furthermore, burial of *R. maxima* larvae at increasing depths delayed emergence of adults, which could be associated with the time and energy required for the larvae to dig upward when placed at greater depths. Similar results with significantly lower rates of adult emergence and delayed adult emergence were observed for *C. nasturtii* when larvae were buried at greater depths (Chen and Shelton 2007).

Although this study is important in providing an initial assessment of the burial behaviors of *R. maxima*, a limitation of this study is that the sifted sand used as a substrate may not be representative of the soil that *R. maxima* would encounter in the field. Previous research has shown that substrate properties can have varying effects on immature cecidomyiids. For example, emergence of *C. nasturtii* adults was found to increase with increasing particle size, but soil type did not have a significant effect on distribution of cocoons or emergence of adults (Readshaw 1968, Chen and Shelton 2007). Therefore, more research is needed using different substrates (eg soil types) and particle sizes, as well as evaluation in field settings. In addition, this study was performed using relatively thin vials, which may have restricted horizontal movement of *R. maxima* larvae that would otherwise occur under field conditions.

Tillage can be used in integrated pest management programs to bury and suppress ground-dwelling pests, including other cecidomyiids (Readshaw 1968, Roger-Estrade et al. 2010, Rowen et al. 2020). As emergence of *R. maxima* adults was more affected by burial of cocoons than larvae, attempts to utilize tillage to reduce adult emergence should focus on cocooned life stages of this pest. For instance, *R. maxima* overwinters as larvae in cocoons, so tillage after harvest at the end of the season or before adult emergence the following spring may have a higher chance of consistently burying cocoons. In addition, developmental (ie degree-day) models for *R. maxima* could provide benefit in determining the specific timing and duration of the targeted life stages. For *C. nasturtii*, tillage alone was shown to be ineffective in preventing emergence of adults in the field, which was attributed to tillage distributing pupae throughout the soil rather than burying them all at the maximum possible depth (Chen et al. 2011). Conversely, a study on *M. destructor* showed that some forms of tillage resulted in no adult emergence (Chapin et al. 1992). This suggests that tillage can be effective at preventing the emergence of soil-dwelling pests, but its efficacy varies depending on the species being managed and how deep and uniformly it is buried. Field studies on the effects of tillage on *R. maxima* are needed to confirm whether this potential cultural control tactic could be incorporated into management programs.

This study serves as a foundation for understanding the burial behavior of *R. maxima.* These findings will facilitate further research examining the burial behavior of *R. maxima* under less controlled conditions and how these behaviors can be exploited for management of this pest. With current management tactics for *R. maxima* providing only limited effectiveness or being logistically challenging, cultural control tactics such as tillage deserve attention.
